# Effective immuno-therapeutic treatment of Canine Leishmaniasis

**DOI:** 10.1371/journal.pntd.0011360

**Published:** 2023-05-22

**Authors:** Rafael Antonio Nascimento Ramos, Alessio Giannelli, François Fasquelle, Angelo Scuotto, Didier Betbeder

**Affiliations:** 1 Laboratory of Parasitology, Federal University of the Agreste of Pernambuco, Garanhuns, Brazil; 2 Veterinarian, PhD, Poulpharm BVBA, Izegem, Belgium; 3 Vaxinano SAS, Loos, France; Bernhard Nocht Institute for Tropical Medicine, Hamburg, Germany, GERMANY

## Abstract

**Background:**

Canine Leishmaniasis (CanL) caused by the *L*. *infantum* species is one of the biggest threats to the health of the South American canine population. Chemotherapeutics currently used for the treatment of CanL fail to induce a total parasite clearance while inducing numerous side effects. As CanL is an immunomodulated disease, the use of immuno-treatments should strengthen the deficient immune response of infected dogs. In this study, we evaluated a nasally administered immunotherapy in dogs naturally infected with *L*. *infantum* (stage 2), with both visceral and cutaneous manifestations. Noteworthy, some of them were also infected by other parasites (*E*. *canis*, *D*. *immitis*, *A*. *platys*), what worsen their chance of survival.

**Methodology/Principal findings:**

The treatment was based on 2 intranasal (IN.) administrations of a killed *L*. *infantum* parasite loaded into maltodextrin nanoparticles, which treatment was compared with the classical oral administration of Miltefosine (2 mg/kg) for 28 days, as well as a combination of these 2 treatments. The results showed that two IN administrations significantly reduced the serology, and were at least as efficient as the chemotherapy to reduce the skin and bone marrow parasite burden, as well as clinical scores, and that unlike Miltefosine treatments, this nasally administered nanoparticle vaccine was without side effects.

**Conclusions:**

These results confirm the feasibility of a simple therapeutic immuno-treatment against *L*. *infantum* infected dogs, which is a promising tool for future developments.

## Author summary

Canine Leishmaniasis (CanL) is an important disease affecting dogs in several regions of the world. Of great zoonotic importance, it is caused by the protozoon *L*. *infantum* that is transmitted through the bite of insect sandflies. The treatment of this disease has been considered a challenge and currently, there are no drugs able to promote a complete parasitological cure. In this study, we evaluated an immuno-therapeutic treatment administered intranasally (IN) in dogs naturally infected with *L*. *infantum* at stage 2. Interestingly, two IN administrations were as efficient as 28 administrations of the Miltefosine treatment to reduce the skin and bone marrow parasite burden, as well as clinical scores. Interestingly, the IFAT decreased sharply, and no side effects were observed. These results demonstrate that the immuno-therapeutic treatment herein used may be a promising tool for the treatment of dogs, and consequently the protection of humans.

## Introduction

Leishmaniases are a group of neglected tropical diseases caused by protozoa belonging to the genus *Leishmania* (Kinetoplastida, Trypanosomatidade) [1]. This zoonotic parasite may infect a wide range of vertebrate hosts, such as wild (e.g., carnivores, marsupials, rodents, and bats) and domestic animals, including dogs and cats [2,3]. The occurrence of this parasite overlaps that of its vector, phlebotomine sandflies [4]. Dogs are considered the main reservoir of *L*. *infantum* subspecies in urban areas for transmission to human [5,6], but themselves may succumb to the infection if it is left untreated or they are unprotected [7].

Canine leishmaniasis (CanL) is considered to be an immunomodulated disease, in which the role of the host’s immune response is pivotal in determining its evolution. As *Leishmania* are intracellular parasites, the Th1 immune response induced after the infection, driven by the adaptive response, helps the infected macrophages to destroy the parasite [8,9]. On the other hand, a switch from Th1 to Th2 response is known to support the parasite’s spread to other organs, mainly bone marrow, the spleen, and the liver, thereby sustaining the development of the disease. The restoration of a Th1 response in CanL should therefore enable the immune cells to better clear the parasite from infected cells, but should also induce a long-term protection through memory T cells [10].

Long-term treatment of the disease with allopurinol, or a combination of allopurinol with meglumine antimoniate or Miltefosine, is currently widely performed for the management of CanL, but relapse is highly probable [11–13]. Beyond the potential nephrotoxicity of some leishmanicidal drugs, a non-effective treatment may affect the success of canine leishmaniasis prognosis [14]. Hence, several recent studies have assessed the role of immunotherapeutic treatments to reestablish dogs’ cellular immunity and consequently improve parasite control compared to chemotherapeutics [15,16]. Cocktails of Th1 cytokines, e.g., IFN- and IL-2, and anti-Th2-cytokines antibodies such as anti-IL-4 and IL-10, were the subjects of the first studies, and aimed to modulate overall immunity [17]. However, despite encouraging results, their limited efficacy and cost remain a barrier to their widespread therapeutic use; in addition, their pleiotropic action and resultant systemic side effects present a further barrier to their adoption [18]. Therefore, more targeted strategies have been developed, and particularly with vaccine formulations used as therapeutics. These are classically made of a killed parasite or subunit antigens, adjuvanted with immunomodulators, which aim to specifically restore the Th1 memory immune response against the parasite [19].

In this study, we investigated the efficacy of a whole *L*. *infantum* antigens (Ag) loaded into maltodextrin nanoparticles, for the control on CanL in naturally infected animals in Brazil. The immunotherapy was administered intranasally (2 doses IN), either alone or in combination immunotherapy (2 doses)/Miltefosine (28 doses) and compared with the classical Miltefosine treatment.

## Methods

### Ethics statement

The Ethical Committee of Animal Experimentation of the Federal Rural University of Pernambuco approved all procedures herein performed (ECAE: 9759161120). Additionally, all dog owners signed a consent form permitting the collection of biological samples.

### Study area

This study was conducted with animals from different municipalities of the state of Pernambuco, Northeastern Brazil. The area has an average temperature of 28 °C, with a mean maximum of 31 °C (from November to April), a mean minimum of 26 °C (from June to September), mean annual rainfall of 2457 mm, and an average relative humidity of 80%. A humid tropical climate predominates in the study area. Additionally, sandflies as well as and canine and human leishmaniasis have been reported in this area [20,21].

### Enrolment and animal care

Thirty dogs were enrolled, aged from 6 months to 10 years old. No animals were pregnant and all were subjected to routine deworming and basic vaccinations. All were clinically classified as Stage II (moderate disease) according to the LeishVet scale. Diagnosis was performed by immunochromatographic TR-DPP Canine Visceral Leishmaniasis test (Bio-Manguinhos, Fiocruz, Brazil), followed by the visual examination of the amastigote forms of *Leishmania spp* in their bone marrow.

Two weeks before the beginning of the study, all animals were dewormed with a combination of praziquantel, pyrantel pamoate and fenbantel (Endal Plus, MSD, Animal Health, Brazil). In addition, blood was collected by venipuncture of the cephalic vein for hematological profiling. All animals remained in their own houses during the entire study period.

### Immunotherapy preparation

Maltodextrin nanoparticles (NP) were prepared as described earlier [22]. Briefly, 100g maltodextrin was dissolved in 2M NaOH with magnetic stirring at room temperature. Then 4.72 mL epichlorohydrin and 31.08 g GTMA were added to get a dense and cationic gel. The gel was neutralized with acetic acid, and crushed through a high-pressure homogenizer (LM20, Microfluidics, France). The particles thus obtained were then filtered by tangential flow ultra-filtration (AKTA flux 6, GE Healthcare, France) through a 750 kDa membrane (GE Healthcare, France), and 70% DPPG (% weight) was added by mixing in water for 2h to obtain the final NP. Their mean average size was 30 nm and their surface charge + 31 mV.

The immunotherapy was made with a whole inactivated *L*. *infantum* strain (purchased from the National Reference Centre for Leishmaniasis (C.Re.Na.L.) of Palermo) as antigens (Ag). The formulation was made by mixing killed parasites and pre-made nanoparticles without addition of any adjuvants. The association of the antigens to the NP was confirmed by native-PAGE. The dose of Ag was based on protein quantification by micro-BCA assay (Pierce, France), and 100μg Ag were administered per dog.

### Study design and follow up assessments

Thirty animals were randomly assigned to three different groups (10 animals per group): G1 –treated with oral Miltefosine (2mg/kg/days for 28 days); G2 –treated with two doses of intranasally administered (IN) immunotherapy (an initial prime dose, then a boost at week 2, 100μg Ag per dose); G3 –treated by both IN immunotherapy and Miltefosine. The study lasted 12 weeks (W) and was performed, after initial assessment, as followed: i) clinical evaluations: W0, W2, W4, W6 and W12; ii) blood and bone marrow collections for microscopy, qPCR and IFAT: W0, W4 and W12. The figures present results at W0, W4 and W12.

### Sampling and laboratorial procedures

#### Preliminary examinations

On day 0 (D0), each dog was photographed, clinically examined, assessed for ectoparasites, and collared with Scalibor (4% Delthametrin) (MSD, Animal Health, Brazil). All data, including sex, age, weight, and clinical signs were recorded in individual files. The clinical status of each animal was determined by a score based on systemic, dermatological, and ocular signs (S1 Table). In addition, animals were screened using a qualitative ELISA test (SNAP 4DX Plus, Idexx Laboratory, Westbrook, Maine, USA) according to the manufacturer’s instructions. This test was able to detect *Dirofilaria immitis* antigens and antibodies against *Anaplasma* spp., *Ehrlichia* spp., and *Borrelia burgdorferi*.

It was noteworthy that most dogs had co-infections (revealed by the SNAP analyses) thus making it difficult to report with total accuracy their exclusively Leishmania-related symptoms: in the Miltefosine group, 8/10 of the dogs were infected by *Ehrlichia canis*, and 1/10 infected by *D*. *immitis*; in the IN immunotherapy group, 9/10 of the dogs were infected by *E*. *canis*, 2/10 infected by *D*. *immitis* and 1/10 infected by *Anaplasma platys*; in the combined treatment group, 8/10 were infected by *E*. *canis*, and 4/10 infected by *D*. *immitis* (Table 1).

**Table 1:** Summary of the co-infections observed in each group.

Group	Co-infection
Miltefosine	*E*. *canis* (80%) *D*. *immitis* (10%)
IN immunotherapy	*E*. *canis* (90%) *D*. *immitis* (20%) *A*. *platys* (10%)
Milt + IN immunotherapy	*E*. *canis* (80%) *D*. *immitis* (40%)

#### Sampling

At each follow up, blood samples were collected by venipuncture of the cephalic vein (8 mL) and stored into two tubes, with and without anticoagulant (Ethylenediaminetetraacetic acid—EDTA). Afterwards, bone marrow (200–400 μL) was collected by puncture of the sternum bone: microscopic slides were prepared and the remaining material stored in plastic tubes for subsequent molecular assessment. Finally, a skin scraping was performed and the material placed in microscopic slides for later analysis. The material was collected through the scrapping of the inner edge of the ulcerations.

At the beginning of the study, all animals were positive for the presence of amastigote forms of *Leishmania* sp. in the bone marrow (BM) after microscopical examination, as expected. The identification of *L*. *infantum* was further achieved by molecular examination (PCR and sequencing, as described below). Regarding the presence of *Leishmania* parasites in the skin, 6/10 dogs were positive in the Miltefosine group, 9/10 in the IN immuno-treatment group and 6/10 in the combined treatments group.

#### Hematology and biochemistry

White blood cell (WBC), red blood cell (RBC), platelet counts, hemoglobin (Hb) and hematocrit (Hct) levels were all determined using a COULTER Hematology Analyzer. Erythrocyte index [mean corpuscular volume (MCV), mean corpuscular hemoglobin (MCH), and mean corpuscular hemoglobin concentration (MCHC)] were calculated according to [23]. The determination of urea, creatinine, albumin, and total protein were performed using the LabTest Liquiform Kit (LabTest, Lagoa Santa, Brazil) and a semi-automatic biochemical analyzer at D0. All slides (bone marrow and skin scraping) were stained using the Panótico Rápido Kit (Laborclin, Pinhais, Brazil) and viewed under an optical microscope (40 × and 100 ×). Results are available in S2 Table.

#### Serologic analysis

Serum samples were examined at W0, W4 and W12 using an immunochromatographic test (TR-DPP Canine Visceral Leishmaniasis, Biomanguinhos, Fiocruz, Brazil) to detect antibodies against *L*. *infantum*. In addition, the Immunofluorescence Antibody Test (IFAT) was performed at the same time-points. Briefly, slides previously impregnated with antigen (promastigotes of *Leishmania sp*.) were used. Sera were serially diluted and tested to establish the maximum reaction titer, starting at a dilution of 1:40. The samples were placed over the antigen in the slides and incubated in a moist chamber at 37o C for 30 minutes. The slides were washed three times in phosphate buffer saline (PBS) and incubated with anti-dog IgG serum conjugated for fluorescein isothiocyanate (KPL, USA) diluted at 1:30 in PBS containing 1 mg% of Evan’s Blue. Finally, each slide was washed in PBS, covered with buffered glycerin and a cover slip, and examined on a fluorescent microscope. Positive and negative controls were included on each slide [24].

#### PCR analysis

Genomic DNA from bone marrow and skin was extracted using the (Wizard Genomic DNA Purification Kit, Promega, Brazil) following the manufacturer’s instructions. Real-time PCR for detecting and quantifying kinetoplast minicircle DNA was performed using the primers LEISH-1 (5’AACTTTTCTGGTCCTCCGGGTAG-3’) and LEISH-2 (5’-ACCCCCAGTTTCCCGCC-3’), and the TaqMan-MGB probe (FAM-5’-AAAAATGGGTGCAGAAAT-3’-non-fluorescent quencher-MGB), as described by Francino et al. [25]. All assays were carried out in triplicate, with a negative control (DNA of a dog from a non-endemic area) and positive control (DNA from the bone marrow of a dog naturally infected by *L*. *infantum*) included in each run.

To confirm the identity of *L*. *infantum* samples were also amplified by conventional PCR using the primers (MC1: 5′-GTTAGCCGATGGTGGTCTTG-3′and MC2: 5′CACCCATTTCGATTTTG-3) following the protocol described by Cortes at al. [26]. The amplifications were viewed in a 1% agarose gel by electrophoresis and a UV transilluminator. Then, the amplified fragments were purified using ExoSAP-IT PCR Product Cleanup Reagent (Applied Biosystems) and sequenced in both directions using the Sanger method in an automatic sequencer ABI 3130 (Applied Biosystems). The chromatograms were analyzed using BioEdit v.7.2.5 software [27] and consensus sequences were submitted to BLASTn search [28] to determine the sequence identity, based upon comparisons with orthologous sequences available in the GenBank database. After sequencing and BLASTn searches, significant identity greater than 99% was observed between the consensus sequence obtained in the present study (OL350822 and OL350823) and *L*. *infantum* sequence DNA available from Genbank database.

Additionally, DNA from skin samples were also tested for species of the subgenus Viannia (including *L*. *braziliensis*) using the following primers: B1 − (5’- GGG GTT GGT GTA ATA TAG TGG—3’) and B2 − (5’- CTA ATT GTG CAC GGG GAG G– 3) [29] and all of them scored negative.

### Data analysis

Comparisons between 2 groups (n < 30) were analyzed by two-tailed Mann-Whitney test for unpaired data. Comparisons between 3 groups (n < 30 per group) were analyzed by Kruskal-Wallis variance comparison test for non-parametric data. A *p* value *<* 0.05 was considered to be statistically significant. Data were analyzed with Prism Software (GraphPad Software Inc. 8.4.2).

## Results

### Study schedule and dogs’ characteristics at enrollment

**Fig 1:**
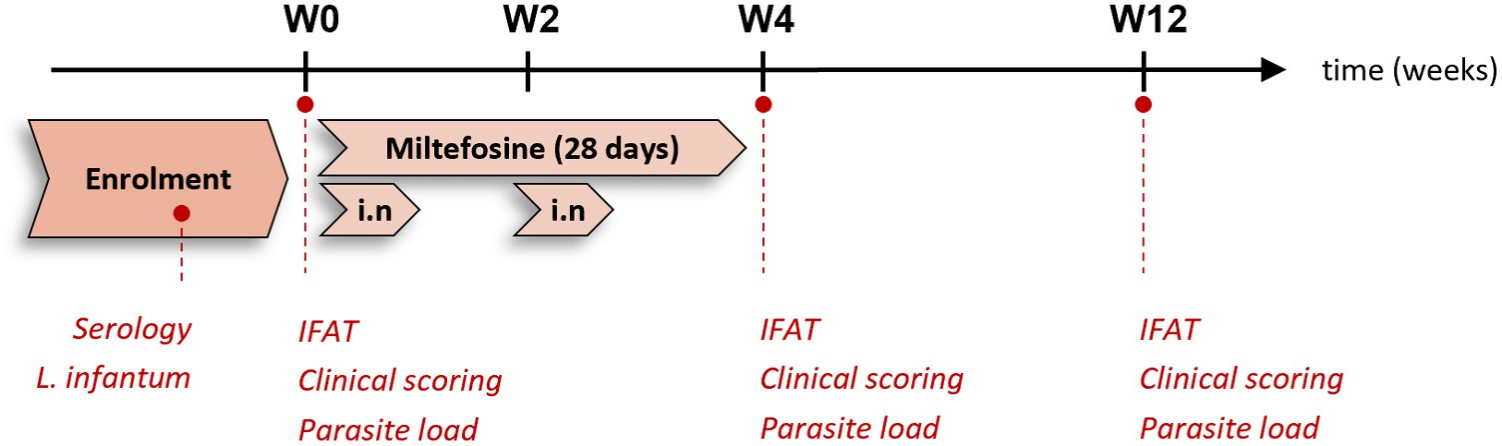
Study schedule with the main parameters assessed in each time-point.

The study schedule is shown in Fig 1.

The three groups were relatively homogeneous in terms of age and of weight at the beginning of the study, with means of 3.6 years old and 15.3 kg in the Miltefosine group, 4.5 years old and 14.7 kg in the IN-immunotherapy group, and 4.7 years old and 13 kg in the combination group (Table 2). Concerning the sex of the animals, males predominated in the combination group (n = 6), followed by the Miltefosine group (n = 3) and by the IN-immunotherapy group (n = 2).

**Table 2:** Sex repartition, mean weight and age of the dogs at the beginning of the study (mean ± SD).

	Sex (M/F)	Weight at W0 (kg)	Age at W0 (year)
Miltefosine	3/7	15.3 ± 4.2	3.6 ± 1.6
IN immunotherapy	2/8	14.7 ± 4.6	4.5 ± 2.6
Milt + IN immunotherapy	6/4	13 ± 4.7	4.7 ± 1.8

The enrolled dogs were all at a stage 2 infection according to the Leishvet classification, with low to high antibody titers and diffuse clinical signs (Leishvet.org). Moreover, they all had kidney malfunction: 100% had hypoalbuminemia, 26/30 had hyperglobulinemia and 29/30 dogs had an albumin/globulin ratio < 1 (S2 Table). At this stage of infection, the clearance of the parasite and the possibility of a total recovery still exists. However, they had an advanced stage of infection (Stage 2 subclass b) as 28/30 of the animals had a creatinine level < 1.4mg/dL (S2 Table).

### Safety

The treatments’ safety was evaluated immediately after the administration and throughout the study. In the Miltefosine group two animals presented an episode of vomit, and one also had diarrhea soon after administration of Miltefosine; in the combination group, one animal exhibited an intense itching, most likely related to the use of the repellent collar. One animal treated with Miltefosine died after W2 follow-up due to a kidney failure, and another one was euthanized at W8 because of a concerning disease progression, despite the treatment. Moreover, one dog treated with IN immunotherapy died most likely due to a snake bite, and one dog receiving the combination died at W8 due to a *D*. *immitis* co-infection (S1 Report).

### IFAT titers

**Fig 2:**
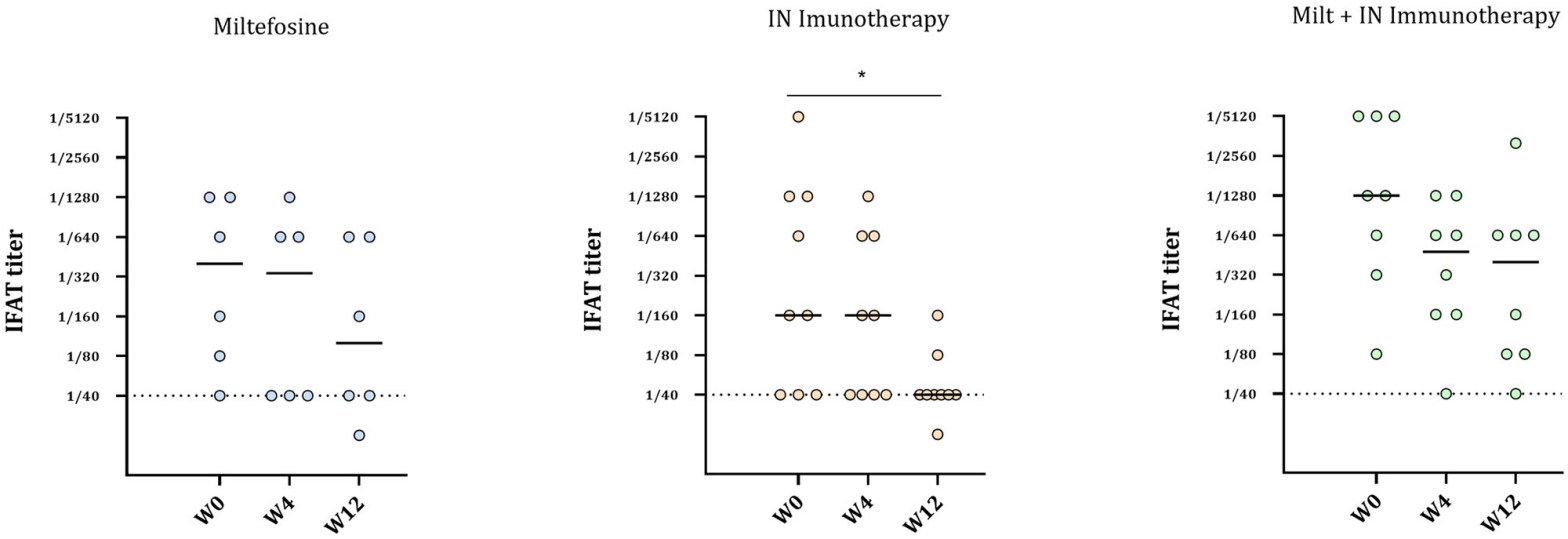
*L*. *infantum* IFAT titers of the dogs in the 3 groups at W0, W4 and W12. Plasma samples were collected at each time point from dogs in the Miltefosine, IN immunotherapy, and Miltefosine + immunotherapy groups. Serology from two dogs in the Miltefosine group and one in the combination group could not be performed at W12. Two dogs died in the Miltefosine group before W12, and two others could not be sampled at W0. One dog in the immunotherapy group and two dogs in the combination group died before W12. Solid lines represent the median values, and dash lines show the IFAT threshold = 1/40. * p < 0.05.

At the beginning of the study, and after randomization, dogs in the Miltefosine group had a median IFAT value of 1/400 (mean = 1/580), dogs from the IN-immunotherapy group had a median value of 1/160 (mean = 1/973) while in the combination IN immunotherapy + Miltefosine group, the IFAT median value was 1/1280 (mean = 1/2370) (Fig 2).

At the end of the study, only the dogs treated by the IN immunotherapy exhibited a significant decrease in IFAT with a median value of 1/40 (mean = 1/56, *p* < 0.05), with one negative dog. These results thus suggest a reduction of the infection. Dogs treated with Miltefosine also had an IFAT decrease, with a median value of 1/100 (mean = 1/257) but without significancy (*p* = 0.26), despite one dog becoming negative. Finally, dogs treated with the combination of the Miltefosine and the immunotherapy had a noticeable, but not statistically significant decrease in the antibody titer (med = 1/400, mean = 1/685, *p* = 0.066), suggesting no synergistic effect between the two treatments.

### Clinical scoring

**Fig 3:**
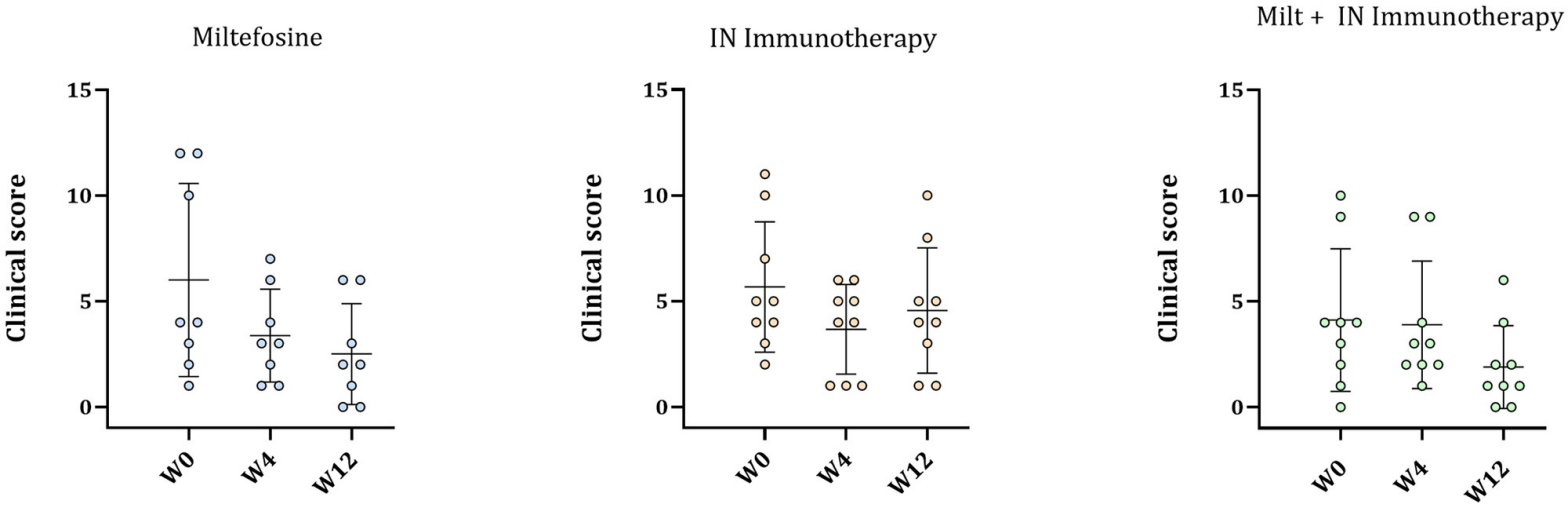
Clinical scores of the dogs in the 3 groups at W0, W4 and W12. The evaluations were made by a veterinary practitioner at the owners’ homes. Horizontal lines represent the mean values ± SD.

The evolution of *L*. *infantum* infection was also monitored by clinical scoring, based on systemic, dermatological, and ocular signs (S1 Table). This clinical study reflects *L*. *infantum* infection associated to co-infections. At the beginning of the study, the dogs in each group had similar clinical scores, with a mean value of 6, 5.7 and 4.1 in the Miltefosine, IN immunotherapy and Miltefosine + IN immunotherapy groups respectively (Fig 3). After three months (W12), a gradual but not statistically significant reduction was observed in Miltefosine group, with mean values of 3.4 at W4 and 2.5 at W12 for dogs. Dogs receiving Miltefosine + IN Immunotherapy had a similar reduction trend, with a score of 3.9 at W4 and 1.9 at W12. Finally, dogs receiving the IN Immunotherapy had a decrease at W4 with a score of 3.7 but a slight increase (mean value: 4.6) was observed at W12.

### Infection in bone marrow and skin

**Fig 4:**
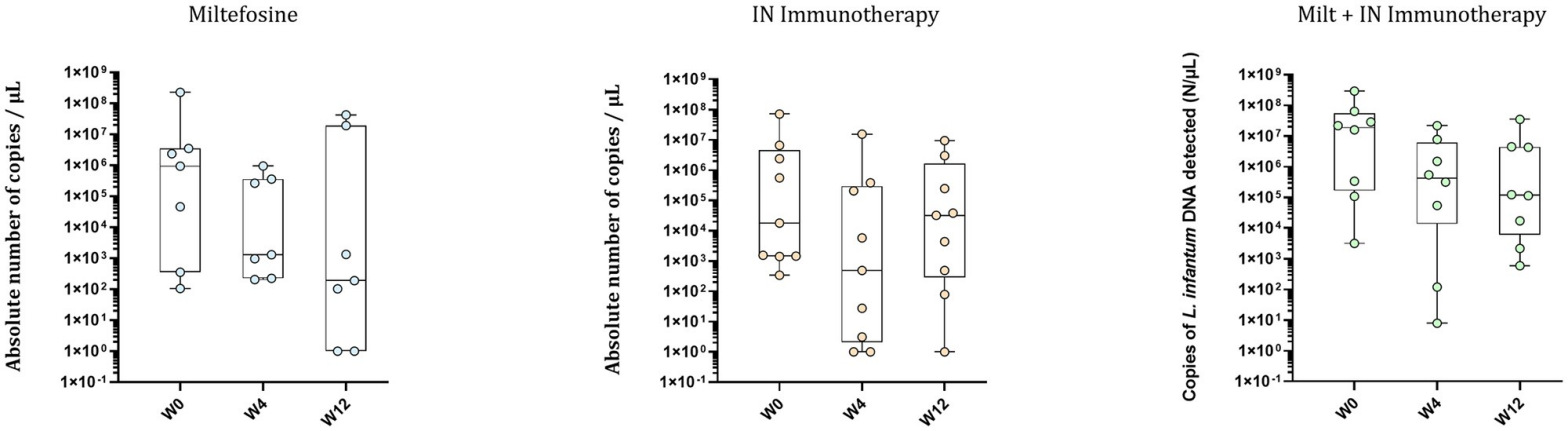
Bone marrow infection: Absolute number of *L*. *infantum* DNA copies per μL (N/μL), analyzed by PCR from BM samples of the dogs in the 3 groups, at W0, W4 and W12. One dog in the Miltefosine group and one in the combination group could not be sampled. Circles represent raw data, and horizontal lines represent the median values. Null values were plotted at 1 N/μL due to logarithmic scale.

CanL infection was surveyed by real-time PCR on bone marrow samples of each dog (Fig 4).

At the beginning of the study, the number of *L*. *infantum* DNA copies per μL (N/μL) in BM were broadly distributed in all groups, with a median of 9.4 x 10^5^ N/μL in the Miltefosine group, 1.8 x 10^4^ N/μL in the IN-immunotherapy group and 1.9 x 10^7^ N/μL in the combination group. After 3 months, the infections generally decreased (though not significantly) particularly for dogs treated with the combination (med = 1.2x10^5^ N/μL, *p* = 0.13), and a tendency also appeared in the Miltefosine group with two dogs which had a clearance of the parasite infection (med = 192 N/μL). The median parasite load remained constant for dogs receiving only IN immunotherapy (med = 3.2 x 10^4^ N/μL), despite one dog appeared to be cured of the infection.

**Fig 5:**
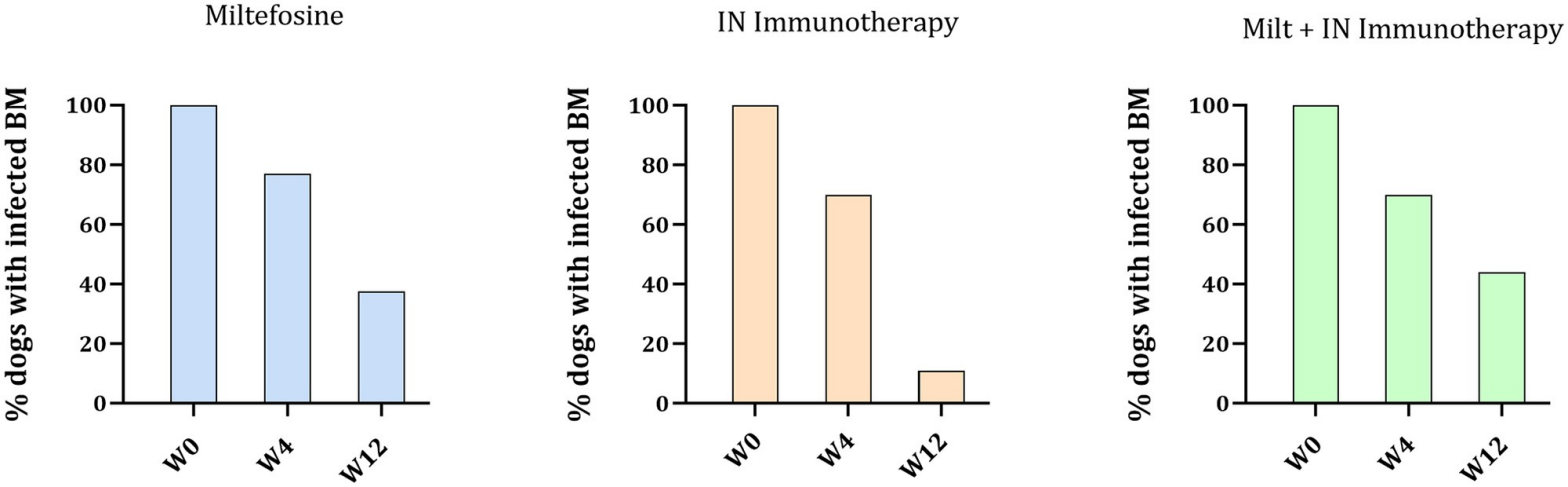
Bone marrow infection: Presence or absence of parasite (%) observed by microscopy from BM samples of the dogs in the 3 groups, at W0, W4 and W12.

The presence of the parasite in bone marrow was also determined by microscopy of the BM samples throughout the study. With this technique, it was shown that 100% of the dogs were positive at the beginning of the study, as expected and regardless of the group. However, after 3 months, 37.5% of dogs treated with the Miltefosine and 44% of dogs receiving the combination were still positive, while only 11% of the dogs treated with the immunotherapy had observable parasites (Fig 5).

**Fig 6:**
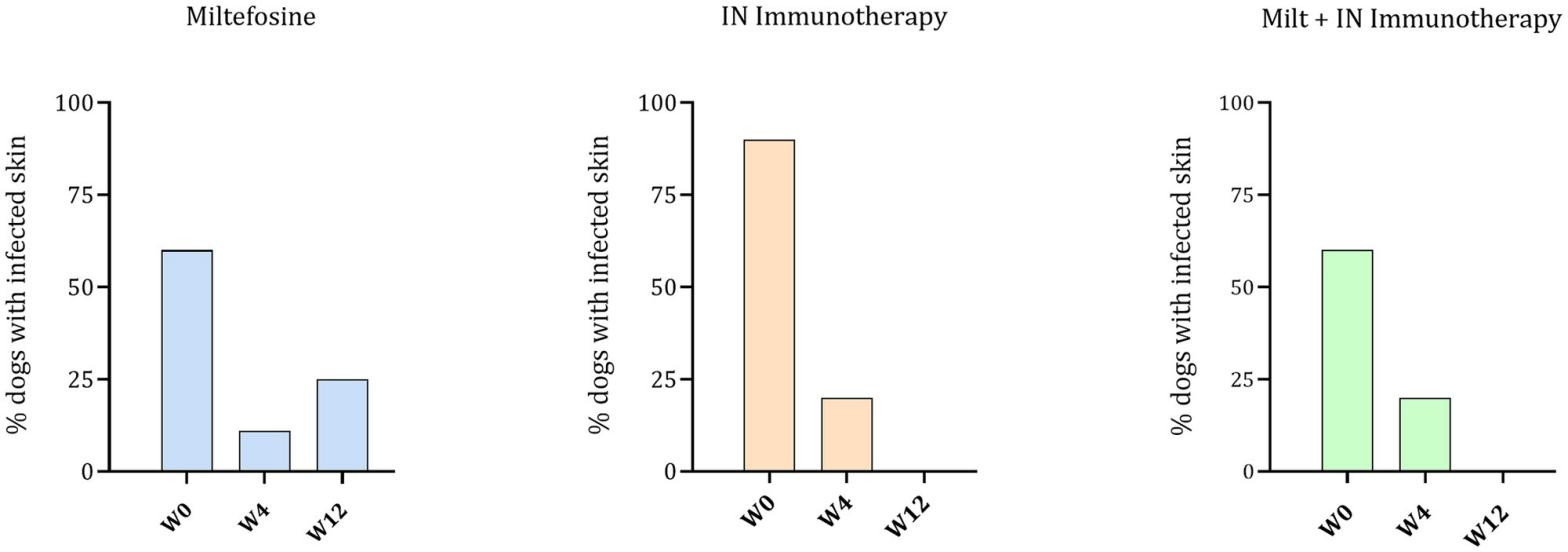
Skin infection: Presence or absence of parasite (%) observed by microscopy from skin samples of the dogs in the 3 groups, at W0, W4 and W12.

Finally, the presence of the parasite in the skin was determined by microscopy after skin scraping, and the percentage of positive dogs was determined (Fig 6).

At the beginning of the study, 60%, 90% and 60% dogs had a visible presence of the parasite in the Miltefosine, IN immunotherapy and in the combination group respectively. However, after 3 months, 20% dogs treated with the Miltefosine were still positive for *L*. *infantum* infection, while all the dogs that received the IN immunotherapy or the combination were negative under microscopic evaluation. Using microscopy, then, the IN immunotherapy seems to reduce the infection in the BM and the skin, whether administered alone or in combination with Miltefosine.

## Discussion

Visceral leishmaniasis is the most severe form of *Leishmania* infection for humans and is fatal in 95% of cases if not treated. As it touches particularly poor populations, this disease was listed by the World Health Organization as a neglected tropical disease and became one of the targets of the Drug for Neglected Diseases Initiative (DND*i*). Indeed, cases of human visceral Leishmaniasis have increased worryingly in recent decades in South America with higher incidence in urban areas, but also present in Europe and in the USA due to the spread of sand fly colonization related to the global warming, and the multiplication of natural reservoir hosts, particularly dogs [13,30].

Despite significant efforts in the development of prophylaxis (insecticide spraying, repellent collars, prophylactic vaccines) and chemotherapies against Leishmania species since the 90’s, none have resulted in a cure for CanL, either due to high treatment costs, deleterious side effects or the development of parasite resistance to the treatment [6]. This current study was performed in field conditions, with domestic dogs who lived and were treated at their home. Thus, the clinical improvement was also dependent of the owner care, with less control of the parameters that may influence the recovery of studied animals. Noteworthy, the dogs were co-infected by other pathogens, mainly *E*. *canis*, *D*. *immitis* and *A*. *platys*, what worsened that overall dog’s health. Because of these conditions, some dogs (n = 2) died before the end of the study due to factors unrelated to leishmaniasis or the treatments: one dog in the IN-immunotherapy group died most likely due to a snake bite, and one of the Miltefosine + IN immune-treatment group died of *D*. *immitis* infection leading to a cardiorespiratory failure. These losses may have impaired the statistical power of the analyses and reduced the possibility of significant statistical differences in the study.

The IN immunotherapy was compared to the Miltefosine treatment which is commonly used in Brazil to treat *Leishmania*-infected dogs. The safety of the different treatment was evaluated from the first administration and throughout the study. Side effects were reported for dogs receiving Miltefosine, with cases of vomiting and diarrhea, and also worsening kidney malfunctions, which have all been described in previous studies [16,31]. On the contrary, no side effect was reported after the initial nasal administration of the immunotherapy, nor thereafter during the study, except a nasal discharge in two dogs, a reaction that disappeared quickly afterwards.

Among the markers evaluated to monitor Leishmaniasis recovery, serological markers and clinical scoring are two of the most representative of the dogs’ overall health [32]. Indeed, under field conditions and in dogs infected by multiple parasites, their serological status might be more specific than the clinical scoring to evaluate the efficiency of a treatment. Here, IFAT and clinical scoring were evaluated in parallel. If both the Miltefosine and the IN immunotherapy helped in reducing the IFAT over the 12 weeks’ treatment, this decrease was significant only for the IN-immunotherapy treatment, with 2/9 dogs above the threshold (Fig 2). However, no synergistic effect was observed on dogs receiving the two treatments, perhaps due to the toxicity of Miltefosine toward immune cells [33]. Two doses of IN immunotherapy were more efficient than a treatment with Miltefosine in aiding recovery from the infection. All the groups showed a decrease in clinical score though in none of the three treatment groups was this decrease significant; a slight increase was also observed in the IN Immunotherapy group from W4 to W12. In the literature, it appears that an IFAT decrease should be directly related to a decrease of the scoring. However, in the present study, most likely the field condition (co-infections) might have impaired the clinical evaluation. Noteworthy, the development of an ELISA analysis in parallel to the IFAT could also improve the evaluation of the immunotreatment’s efficacy.

The evolution of the infection was also monitored by measuring the parasite burden of the visceral infection using qPCR and microscopy, as reported in similar studies [34]. These measurements were made in bone marrow, and in skin (only by microscopy) to assess the cutaneous dissemination. It is important to use a mix of methods due to their mid sensitivity (73% for qPCR, 52–85% for microscopy) [35]. In the BM, a broad variation of the infection in each group was observed at the beginning of the study by qPCR analyses (from 10^2^ to 10^9^), what is coherent with the field study conditions, where animals have different infection histories contrary to experimentally infected animals. Still, a decrease in the median parasite burden for dogs treated with Miltefosine and with the IN immunotherapy + Miltefosine combination, while parasite burden remained unchanged for dogs receiving the IN Immunotherapy (despite one dog becoming negative). Noteworthy, PCR tests are very sensitive. In this case the detection of small concentrations of DNA does not necessarily indicates the presence of live parasites. However, by microscopy, 89% of the dogs treated with the immunotherapy were found to be negative after three months, while 37.5% remained positive in the Miltefosine group and 44% in the combination group. In the skin, after 3 months treatment, 100% of the dogs that received the IN immunotherapy or the IN immunotherapy + Miltefosine combination were negative under microscopic evaluation, while 20% dogs treated with the Miltefosine were still positive for *L*. *infantum* infection. Despite the low sensitivity of microscopic techniques, the absence of *L*. *infantum* amastigote in the skin is noteworthy, as it may play an important role as source of transmission by sandflies vectors [36].

Taken together, these data suggest that two nasal doses of immunotherapy seem to be as efficient as the Miltefosine in decreasing the parasite burden in the BM but are more efficient at eliminating skin infection. This study thus shows that nasal treatment can be used as a simple, safe, and effective immunotherapy to treat canine leishmaniasis.

## Supporting information

S1 Table Clinical status of each animal based on score determination. (XLSX)

S2 Table Biochemistry data. (XLSX)

S1 Report Necropsy reports. (PDF)
