# Supplementary material for: Effective immuno-therapeutic treatment of Canine Leishmaniasis
Source: PLoS Negl Trop Dis. 2023 May 22;17(5):e0011360. doi: 10.1371/journal.pntd.0011360 (PMC10237639; doi:10.1371/journal.pntd.0011360)
Supplement: S1 Report — (PDF) [file pntd.0011360.s003.pdf]

Federal Rural University of Pernambuco
Department of Veterinary Medicine
Pathology Area

TRANSLATION OF THE OFFICIAL REPORT
(By Rafael Ramos)
Result of necroscopic examination
N°: 5488

Animal: Cara Velha
Species: Canine **Breed**: Crossbreed **Sex**: Male
Age: 5 years old **Hair**: Short
Responsible: Rafael Antônio do Nascimento Ramos
Address: Rua da Esperança, 129, Santo Antônio, Garanhuns
Veterinary: Rafael Antônio do Nascimento Ramos

Clinical and laboratorial history of the animal: *Leishmania infantum* positive dog diagnosed in February 2021. The confirmation of infection was achieved by means of microscopy (bone marrow), serology (ELISA and IFAT), and molecular (qPCR) methods. On April the animal started the treatment for CVL with Miltefosine and an intranasal formulation. The animal remained stable, but at the end of May started to present clinical signs suggestive of renal disease. On May 27 the animal was attended in a private veterinary clinic and two days after died.

Pathological findings: at the external the animal presented bad nutritional status, enophthalmia, pale oral and ocular mucous, presence of loose stools in the perianal region; and the edges of the tongue with a greenish aspect. At the internal examination was observed the coloration of lungs diffuse red, in addition was detected a dark red focal area on the pleural surface of the accessory lobe. The trachea was flattened dorso–ventrally and a foamy fluid was observed inside it. The heart had a focally extensive white area in the right ventricle; and adult nematodes (*Dirofilaria immitis*) were observed in the right ventricular chamber and pulmonary artery. At the evaluation of the abdominal cavity splenomegaly was observed and a slight focal area of fibrosis detected in this organ. Finally, the kidneys had shiny, diffusely dark aspects.

Morphological diagnosis:

- Cachexia
- Pneumonia
- Tracheal stenosis
- Pulmonary edema
- *Dirofilaria immitis* in the right ventricle
- Splenomegaly

Cadaveric changes: hemolytic soak.

Conclusion: Cardiorespiratory failure

Recife, 19 July 2021

Federal Rural University of Pernambuco
Department of Veterinary Medicine
Pathology Area

TRANSLATION OF THE OFFICIAL REPORT
(By Rafael Ramos)

Result of necroscopic examination
N°: 5497

Animal: Irmã da Fox
Species: Canine **Brees**: Crossbreed **Sex**: Female
Age: 2 years old **Hair**: Short
Responsible: Rafael Antônio do Nascimento Ramos
Address: Rua da Esperança, 129, Santo Antônio, Garanhuns.
Veterinary: Rafael Antônio do Nascimento Ramos

Clinical and laboratorial history of the animal: *Leishmania infantum* positive dog diagnosed in February 2021. The confirmation of infection was achieved by means of microscopy (bone marrow), serology (ELISA and IFAT), and molecular (qPCR) methods. On April the animal started the treatment for CVL with an intranasal formulation. The animal was in good condition and suddenly at the end of June died. A suggestive lesion of snakebite was observed in the posterior right paw.

Pathological findings: at the external examination was observed animal with pale mucous, good nutritional status and suggestive lesion of snakebite in the posterior right paw. At the internal examination were observed enlargement of lymph nodes (popliteal). At thoracic cavity a discrete quantity of free bloody liquid was observed and the presence of moderate foamy liquid inside the trachea. Additionally, enlargement of spleen and liver was observed.

Morphological diagnosis

- Anemia
- Pleural effusion
- Pulmonary congestion
- Pulmonary edema
- Splenomegaly
- Hepatomegaly
- Suggestive lesion of snakebite

Cadaveric changes: hemolytic soak

Conclusion: suggestive of snakebite

Recife, 15 July 2021

Federal Rural University of Pernambuco
Department of Veterinary Medicine
Pathology Area

TRANSLATION OF THE OFFICIAL REPORT
(By Rafael Ramos)

Result of necroscopic examination

N°: 5491

Animal: Malhado
Species: Canine **Breed**: Pitbull **Sex**: Male
Age: 2 years old **Hair**: short, black and yellow
Responsible: Rafael Antônio do Nascimento Ramos
Address: Rua da Esperança, 129, Santo Antônio, Garanhuns.
Veterinary: Rafael Antônio do Nascimento Ramos

Clinical and laboratorial history of the animal: *Leishmania infantum* positive dog diagnosed in February 2021. The confirmation of infection was achieved by means of microscopy (bone marrow), serology (ELISA and IFAT), and molecular (qPCR) methods. On April the animal started the treatment for CVL with Miltefosine and Prednisolone. At beginning of the treatment the animal presented apathy, weight loss, difficulty to move, conjunctivitis and scleritis. On the first 15 days of treatment the animal improved and a slightly weight body gain was observed. At the end of the treatment (28 days) the animal worsened, hence Prednisolone was recommended again, but no improvement was observed and an intense weight loss was observed. The animal presented normal levels of creatinine and no significant alterations on the count blood cell. For decision of the owner the dog was euthanatized (Ketamine, Acepromazine, Propofol and Potassium chloride were used in this procedure).

Pathological findings: at the external examination the presence of ectoparasite were observed. Animal with pale mucous, intense enophthalmia and bad nutritional status with muscle atrophy. At the internal examination were observed enlargement of lymph nodes (pre–scapular, scapular, sub–mandibular and popliteal). At thoracic cavity a discrete quantity of free serous liquid was observed, lung with red color and hypercreptating left cranial lobe. In addition, moderate presence of foamy liquid inside the trachea was detected. The esophagus presented a slight dilation in the distal third portion. The heart had enlargement of the left ventricular chamber and reduction of the respective wall; right ventricle had loss of shape of the trabeculae meat. In the evaluation of the abdominal cavity, no adipose tissue was observed, and spleen was enlarged. Finally, invagination of the antrum region in the body region of stomach was observed.

Morphological diagnosis:
- Cachexia
- Anemia
- Pleural effusion
- Pulmonary congestion
- Pulmonary edema
- Bilateral ventricular cardiac dilatation
- Splenomegaly
- Gastric intussusception

Cadaveric changes: hemolytic soak.

Conclusion: animal euthanized
